# Development and Analysis of the Physicochemical and Mechanical Properties of Diorite-Reinforced Epoxy Composites

**DOI:** 10.3390/polym13152421

**Published:** 2021-07-23

**Authors:** Amirbek Bekeshev, Anton Mostovoy, Yulia Kadykova, Marzhan Akhmetova, Lyazzat Tastanova, Marina Lopukhova

**Affiliations:** 1 Laboratory “Polymer Composites”, K. Zhubanov Aktobe Regional State University, Aliya Moldagulova Avenue 34, Aktobe 030000, Kazakhstan; amirbek2401@gmail.com; 2 Laboratory “Modern Methods of Research of Functional Materials and Systems”, Yuri Gagarin State Technical University of Saratov, Polytechnichskaya St., 77, 410054 Saratov, Russia; 3 Department “Electricity and electrical engineering”, Yuri Gagarin State Technical University of Saratov, Polytechnichskaya St., 77, 410054 Saratov, Russia; Kadykova06@yandex.ru; 4 Department “Physics”, K. Zhubanov Aktobe Regional State University, Aliya Moldagulova Avenue 34, Aktobe 030000, Kazakhstan; majiko.a@gmail.com; 5 Department “Chemistry and chemical technology”, K. Zhubanov Aktobe Regional State University, Aliya Moldagulova Avenue 34, Aktobe 030000, Kazakhstan; lyazzatt@mail.ru; 6 Department “Economics and Humanitarian Sciences”, Yuri Gagarin State Technical University of Saratov, Polytechnichskaya St., 77, 410054 Saratov, Russia; mlopuhova@yandex.ru

**Keywords:** epoxy oligomer, modification, plasticizer, filler, diorite, physicochemical and mechanical properties

## Abstract

The aim of this paper is to study the effect of a polyfunctional modifier oligo (resorcinol phenyl phosphate) with terminal phenyl groups and a dispersed mineral filler, diorite, on the physicochemical and deformation-strength properties of epoxy-based composites. The efficiency of using diorite as an active filler of an epoxy polymer, ensuring an increase in strength and a change in the physicochemical properties of epoxy composites, has been proven. We selected the optimal content of diorite both as a structuring additive and as a filler in the composition of the epoxy composite (0.1 and 50 parts by mass), at which diorite reinforces the epoxy composite. It has been found that the addition of diorite into the epoxy composite results in an increase in the Vicat heat resistance from 132 to 140–188 °C and increases the thermal stability of the epoxy composite, which is observed in a shift of the initial destruction temperature to higher temperatures. Furthermore, during the thermal destruction of the composite, the yield of carbonized structures increases (from 54 to 70–77% of the mass), preventing the release of volatile pyrolysis products into the gas phase, which leads to a decrease in the flammability of the epoxy composite. The efficiency of the functionalization of the diorite surface with APTES has been proven, which ensures chemical interaction at the polymer matrix/filler interface and also prevents the aggregation of diorite particles, which, in general, provides an increase in the strength characteristics of epoxy-based composite materials by 10–48%.

## 1. Introduction

In the modern world, requirements for performance properties for various materials and products obtained from them are constantly increasing and it is possible to provide them by selecting raw materials and the technological parameters of production [1,2,3]. 

The combining or physicochemical modification of various materials that make up epoxy compositions makes it possible to directly control the most important properties of epoxy composites. The addition of plasticizers provides the elasticity of polymeric materials and can change their glass transition temperature [3,4,5]. The addition of fillers ensures an increase in the strength of epoxy composites and imparts specific physical and chemical properties to them [6,7,8,9]. The effect of the addition of a filler on the properties of the polymer is determined by many factors: the chemical nature of the polymer and filler, the nature of the filler surface, the size and shape of its particles, the ability to form their own structures, a change in the conformational set of macromolecules and the structure of the polymer itself. The added fillers with different quantitative content have different effects on the structure of polymers [10,11,12,13].

It was shown in the work [14,15] that the integration of silica nanoparticles in an epoxy nanocomposite can greatly improve the mechanical properties of the resultant composite materials, including, but not limited to, tensile strength, fracture toughness, impact and fatigue properties.

The authors of [16,17] propose to use technogenic waste from industrial enterprises—fly ash, which is a by-product of coal burning at thermal power plants—as a filler for epoxy composites. Fly ash was introduced into the epoxy composite in an amount of 10–50 vol.%. The studies carried out have shown that, as the amount of fly ash increases to the critical point (30 vol.%), an increase in tensile strength is apparent, while the compressive strength of the composite continuously increases with an increase in the fly ash con-tent. In addition, it was found that reducing the particle size of fly ash provides the production of epoxy composites with higher physical and mechanical properties.

In work [18], bi-functionalized montmorillonite was used as a filler in epoxy compo-sites. Epoxy composites filled with organoclay have higher thermal stability; in addition, the introduction of organoclay provides an increase in the yield of carbonized structures being a physical barrier for the interdiffusion of the oxidizer and combustible gases into the combustion zone, which reduces the flammability of the epoxy composite. Studies have shown that the optimal amount of bi-functionalized montmorillonite is 1 wt.%, which provides an increase in storage modulus, cross-link density and glass transition temperature of epoxy composite.

Granite powder is a promising filler for epoxy composites, the introduction of which not only reduces the cost of composites but also improves their physical and mechanical properties. In work [19], to increase the adhesive interaction at the polymer matrix/filler interface, the granite powder was treated with triethoxymethylsilane. Granite powder was added to the epoxy composition in an amount of 0–60 wt.%. Composites with 50 wt.% of granite powder were found to have maximum mechanical properties in all cases. The treatment of granite powder with a silane coupling agent improves the mechanical prop-erties of epoxy composites based on it and also improves the interfacial bond between the granite powder and the epoxy matrix.

The study of the mechanism of physical and chemical cross-linking processes when various plasticizers and fillers are added into an epoxy binder is an important issue in modern materials science.

Currently, despite a large number of works devoted to the study of the effect of various fillers and plasticizers, there are still insufficiently studied issues related to their in-fluence on the processes of structure formation and the structure and performance char-acteristics of polymer composite materials, which predetermines the direction of research in this work.

The aim of this paper is to study the effect of a polyfunctional modifier oligo (resorcinol phenyl phosphate) with terminal phenyl groups and a dispersed mineral filler, diorite, on the physicochemical and deformation-strength properties of epoxy-based compo-sites.

## 2. Materials and Methods

### 2.1. Materials

We used ED-20 epoxy resin (GOST 10587-93) manufactured by CHIMEX Limited (St. Petersburg, Russia) as a binder for the preparation of polymer composite materials, and polyethylene polyamine (PEPA) (TU 6-02-594-85) manufactured by CHIMEX Limited (St. Petersburg, Russia) as a hardener.

Oligo (resorcinol phenyl phosphate) with terminal phenyl groups Fyrolflex (ORPP) manufactured by ICL Industrial Products America Inc. (New York City, NY, USA) was used as a plasticizer and flame retardant.

ORPP is an oligomeric halogen-free plasticizer with flame-retardant properties. Compared to other halogen-free (phosphate) fire retardants, it has low volatility and stability (onset of thermal degradation at +370 °C), which allows its use for modifying most technical plastics. The advantage of ORPP over other bis-phosphates is its lower viscosity, which makes the product easier to handle and improves its technological properties (lower mixing temperature) [6,20].

Table 1 shows the typical properties and specifications of epoxy resin, hardener and ORPP.

The presence of phosphorus (10.7%) as a combustion inhibitor in ORPP ensures the structuring of the epoxy composite when exposed to elevated temperatures, which leads to an increase in the yield of carbonized structures being a physical barrier for the interdiffusion of the oxidizer and combustible gases into the combustion zone, which reduces the flammability of the epoxy composite [6,20,21].

As a filler for epoxy composites, we used finely ground magmatic plutonic rock, diorite, from the Priorskoye deposit (Novorossiysk district of Aktobe region, Kazakhstan). The choice of diorite as a filler for polymer composites is due to its availability; diorite is very actively mined in Chile, Peru, Ecuador, Sweden and Norway. In Kazakhstan and Russia, there are several very large deposits of this rock. In addition, diorite is not only hard, but also viscous, which ensures high wear resistance of the rock. The choice of diorite as a filler is associated not only with its availability but also with a certain chemical composition: the presence of metal oxides (iron oxides, calcium, aluminum and titanium) will allow diorite to be used as a flame retardant for epoxy polymers. Based on the properties of diorite, it can be expected that its introduction into a polymer composition will provide an increase in the physicochemical and mechanical properties of polymer composites.

### 2.2. Functionalization of the Diorite Surface

The diorite surface was functionalized with γ-aminopropyltriethoxysilane (APTES) manufactured by Penta-91 (Russia). For this, 0.5 g of diorite was dispersed in 100 ml of a solution of H_2_O—APTES (95–5) for 10 min using an ultrasonic homogenizer. To increase the solubility of APTES in water, the pH of the mixture was brought to 5 with the gradual addition of acetic acid. We selected an acidic media (CH_3_COOH) in order to increase the level of silanol formation and to decrease self-condensation reactions between the hydro-lyzed silanol groups. To remove the remaining silane compound around the diorite particles, the resulting suspension was centrifuged and washed twice with H_2_O [22]. Then the product was dried at 105 °C in the laboratory oven for 5 h.

### 2.3. Characterization of Diorite

The surface morphology of the samples was studied using a Tescan VEGA 3 SBH scanning electron microscope (Brno, Czech Repuplic). X-ray phase analysis was carried out using an ARL X′TRA diffractometer (CuKα radiation, λ = 0.15412 nm, angle range 2θ 5°–60°). The diffraction patterns were interpreted using the Powder Diffraction File-2 (PDF-2) database of the International Center for Diffraction Data (ICDD) and the Crystallographic Search-Match program, version 3.1.0.2.B. The chemical composition of diorite was determined on an X-ray analytical microprobe-microscope PAM 30-μ. FT-IR spectroscopy was carried out using the Shimadzu IRTracer-100.

The size distribution of diorite particles was determined by laser diffraction in an aqueous medium using a Fritsch Analysette-22 Nanotech analyzer (Fritsch, Germany) in the range of 0.01–1000 μm. Determination of the specific surface area of the samples was carried out using an Quantachrome Nova 2200 surface area and porosity analyzer from the low-temperature nitrogen adsorption.

### 2.4. Preparation of Epoxy Composites

Diorite was added into the plasticized ORPP epoxy composition as a modifying ad-ditive (0.05–0.50 parts by mass) and a filler (50–100 parts by mass). For uniform distribution of the filler in the polymer matrix and preventing its aggregation, ultrasound treatment of the epoxy composition was used (ultrasound exposure parameters: frequency 22 ± 2 kHz, duration 60 min) [6].

The epoxy composition was cured at room temperature for 24 ± 1 h, followed by stepwise heat treatment at 90 ± 5 °C for 2 h and at 120 ± 5 °C for 2 h [6,13,20].

### 2.5. Testing of the Composites

Tests of samples for resistance to tensile and bending loads were carried out on a WDW-5E universal electromechanical testing machine from Time Group Inc., Beijing, China, at a test speed of 5 mm/min for the tensile test and 50 mm/min for the bending test. The bending stress and flexural modulus was determined according to ISO 178: 2019; the tests were carried out on samples in the form of blocks with 4 mm thickness, 10 mm width and a working-part length of 80 mm. The strength and modulus of tensile elasticity was determined according to ISO 527-2: 2012; the tests were carried out on samples in the form of spatulas with 4 mm thickness, 10 mm width and a working-part length of 50 mm.

Compressive strength was determined in accordance with ISO 604: 2002; the tests were carried out on samples in the form of a cube with rib length 30 mm. The impact strength was determined in accordance with ISO 179-1: 2010 using an LCT-50D pendulum impact machine (Beijing United Test Co., Ltd., Beijing, China); the tests were carried out on samples in the form of blocks with 4 mm thickness, 10 mm width and a working-part length of 80 mm.

The Brinell hardness was determined in accordance with ISO 2039-1: 2001 using an HBE-3000A electronic Brinell hardness tester (Beijing United Test Co., Ltd., Beijing, China). The determination of the Vicat heat resistance was carried out according to ISO 306: 2013, method B50–load 50 N; temperature rise rate 50 °C/h.

The surface morphology of the samples was studied using a Tescan VEGA 3 SBH scanning electron microscope (Brno, Czech Repuplic).

Thermal stability of the samples was determined by a thermogravimetric analysis using a Q-1500D derivatograph (MOM, Budapest, Hungary) of the Paulik–Paulik–Erdey system under the following experimental conditions: weighed portion—100 mg, medium-air, heating interval—up to 1000 °C, heating rate −10 °C/min, a relative error that does not exceed 1%.

To determine the mass loss during ignition in air, samples were made with a width 35 ± 1 mm, a length 150 ± 3 mm and a height 4 ± 1 mm. Pre-weighed (accurate to 0.0001 g) samples were suspended vertically in the center of a metal tube so that the end of the sample protruded 5 mm and was 10 mm above the gas burner. A gas burner with a flame height of 40 ± 5 mm was placed under the sample in its center. After 2 min of exposure to the flame, the ignition source was removed, and the sample continued to burn or smolder on its own. After cooling to room temperature, the sample was weighed (with an accuracy of 0.0001 g), and the weight loss was determined as a percentage of the initial sample weight using the formula: Δm = (m_1_ − m_2_)·100/m_1_, where m_1_—sample weight before testing; m_2_—sample weight after testing.

The self-heating temperature of the sample during the curing of the epoxy composition was determined according to the method described in [7].

The degree of curing of epoxy composites was determined by extracting samples of the crushed material with acetone in a Soxhlet apparatus. A sample of finely ground material weighing 1 g was poured with 20 ml of acetone and extracted in the apparatus for 24 h., then the material was dried and the dry residue was weighed with an accuracy of 0.0001 g. The change in weight was calculated by the formula: Δm = (m_1_ − m_2_)·100/m_1_, where m_1_—initial sample weight; m_2_—weight of the sample after extraction and drying. The degree of curing (X) was determined by the formula: X = 100 − Δm.

## 3. Results and Discussion

**Figure 1:**
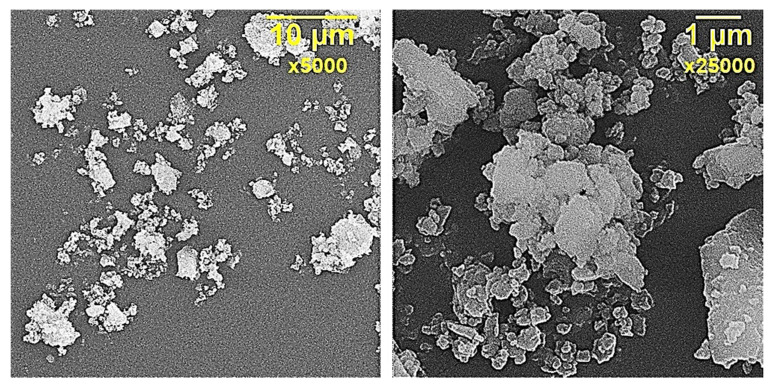
SEM of diorite particles.

According to SEM data, diorite crystals are flattened, tabular to scaly or lamellar, Figure 1.

The study of the chemical composition of diorite has shown that it mainly consists of iron oxides, silicon, calcium, aluminum and titanium, Table 2.

**Figure 2:**
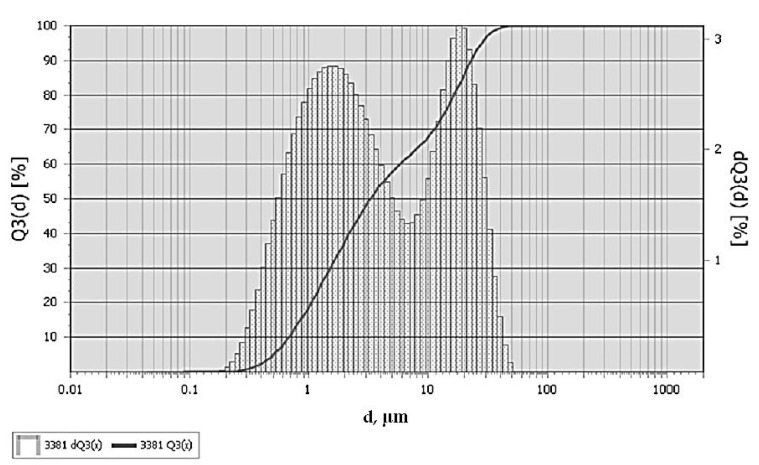
Fractional composition of diorite.

The fractional composition of diorite is represented by particles from 0.2 to 50 μm, with average particle sizes of 1–2 and 20–25 μm, Figure 2.

**Figure 3:**
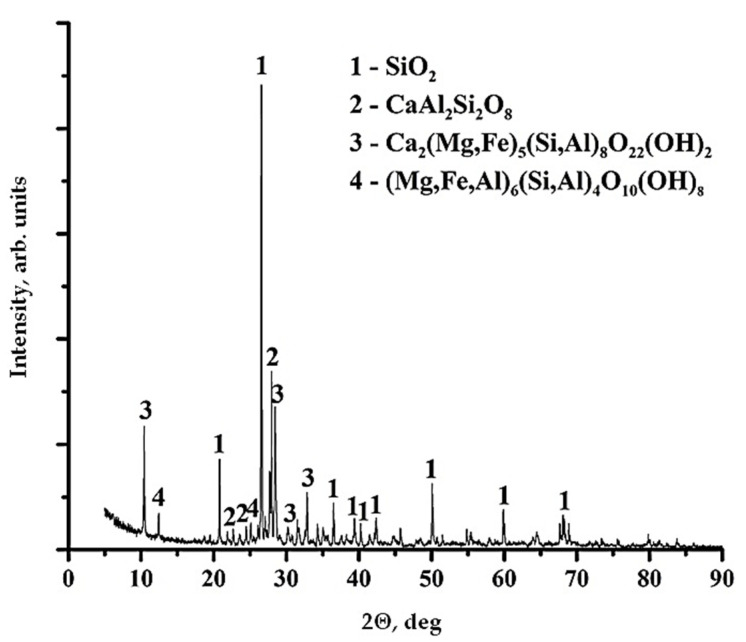
XRF data for diorite.

XRF data have shown that diorite is represented by four different phase structures, in Figure 3.

The specific surface area of diorite particles, determined using a Quantachrome Nova 2200 Surface Area and Pore Size Analyzer by a low-temperature nitrogen adsorption method, is 5.6 m^2^/g.

Thus, the analysis of the structure and specific surface area of diorite has shown that it can be used as a structuring additive and filler for epoxy composites, which should ensure an increase in their performance properties. Pre-drying the filler is not required because the moisture content in diorite is 0.4%.

Based on results of the studies, it has been found that the optimal content of diorite as a structuring additive in the epoxy composition is 0.1 parts by mass, which provides a significant increase in the indicators of physical and mechanical properties: the bending stress increases by 19%; the compressive strength increases by 15%; the strength increases by 80%, and the tensile modulus increases by 31%; the impact strength increases by 100%; the Brinell hardness increases by 20%; all are shown in Table 3.

The optimal content of diorite as a filler is 50 parts by mass, which not only reduces the cost but also increases the strength characteristics of filled epoxy composites: the bending stress increases by 31%, and the modulus of elasticity in bending increases by 3.6 times; the compressive strength increases by 55%; the tensile strength increases by 40%, and the tensile modulus increases by a factor of 2.4; the Brinell hardness increases by 68%, while the impact toughness remains at the level of the unfilled plasticized composite, as shown in Table 3.

The change in the viscosity of the epoxy composition when diorite is introduced into it is shown in Table 4.

**Figure 4:**
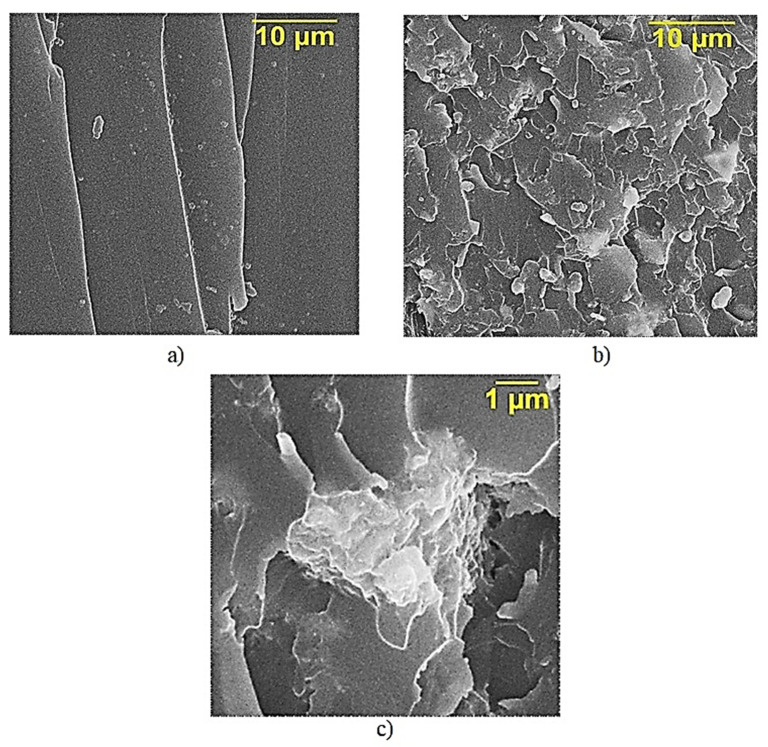
SEM data of samples of epoxy composites, parts by mass: (**a**)—100 ED-20 + 40 ORPP + 15 PEPA (×5000); (**b**)—100 ED-20 + 40 ORPP + 50 Diorite + 15 PEPA (×5000); (**c**)—100 ED-20 + 40 ORPP + 50 Diorite + 15 PEPA (×25,000).

From the presented SEM data, it can be seen in Figure 4 that, by adding diorite, a monolithic structure of the sample is formed and an almost complete absence of aggregates of diorite particles is observed, as shown in Figure 4b. The binder molecules repeat the layer morphology of the filler, as shown in Figure 4b–c, which is apparently one of the facts explaining the significant increase in the properties of epoxy composites in the presence of an active filler, diorite.

The analysis of the literature data has shown that the addition of dispersed fillers affects the kinetic characteristics of the polymerization reaction of the epoxy composition during curing as well as the formation of the phase structure of the composite material [6,13,23,24,25,26,27].

**Figure 5:**
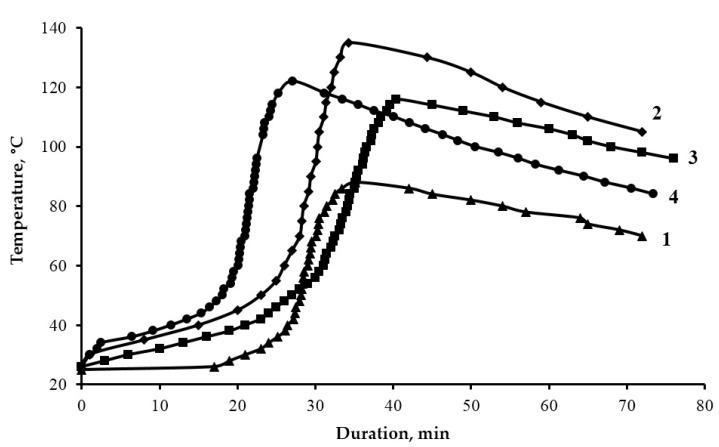
Kinetic curves of the curing process of epoxy composites: 1—100 ED-20 + 40 ORPP + 15 PEPA; 2—100 ED-20 + 40 ORPP + 0.1 Diorite + 15 PEPA; 3—100 ED-20 + 40 ORPP + 50 Diorite + 15 PEPA; 4—100 ED-20 + 40 ORPP + 50 Diorite_(APTES)_ + 15 PEPA.

The analysis of the curing kinetics of epoxy compositions has shown that the addition of diorite (0.1 parts by mass) has an initiating effect on the processes of structure formation, as shown in Figure 5, which is observed in the reduction in the gelation time from 27 to 24 min and the curing time from 38 to 34 min. However, with the addition of diorite as a filler (50 parts by mass), the gelation time and curing time slightly increase (from 27 to 29 min and from 38 to 40 min, respectively), which is apparently due to the high viscosity of the composition and steric hindrances in the curing process, as shown in Table 5.

Thus, in both cases, there is a significant increase in the maximum curing temperature from 88 to 116–135 °C. Besides, the addition of diorite into the epoxy composition provides an increase in the curing degree of the epoxy composite from 90 to 94–98%, which allows the assumption that the diorite particles are additional cross-linking centers.

**Figure 6:**
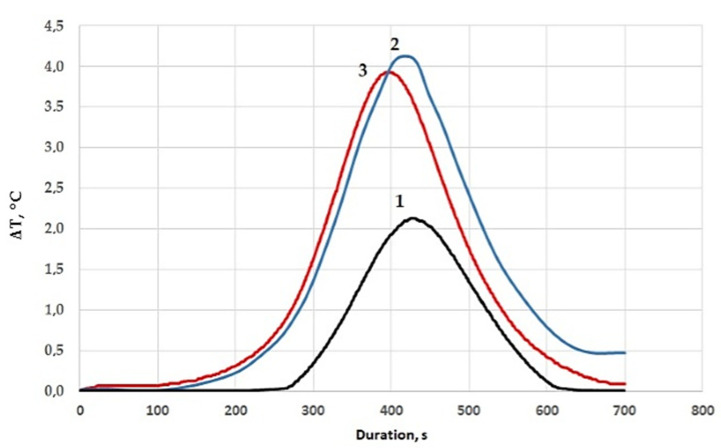
Differential scanning calorimetry of epoxy compositions: 1—100 ED-20 + 40 ORPP + 15 PEPA; 2—100 ED-20 + 40 ORPP + 0.1 Diorite + 15 PEPA; 3—100 ED-20 + 40 ORPP + 50 Diorite + 15 PEPA.

Differential scanning calorimetry data confirmed the results obtained, as shown in Figure 6, in which the introduction of diorite into the epoxy composition provides an increase in the thermal effect of the reaction and a decrease in the temperature of the onset of curing from 46 to 33–36 °C; in addition, an increase in the glass transition temperature of the epoxy composite was noted, as shown in Table 6.

The addition of diorite into the epoxy composition provides an improvement of the physicochemical and thermophysical parameters of epoxy composites, which is observed in an increase in the Vicat heat resistance from 132 to 140–188 °C, as shown in Table 7. Besides, the thermal stability of the composite increases, which can be seen in a shift of the initial temperature of the main stage of destruction to the area of higher temperatures (from 230 to 240–245 °C). It has been found that the thermal destruction of the composite containing diorite increases the yield of carbonized structures from 54 to 70–77%, which prevents the release of volatile thermolysis products into the gas phase, thus leading to a decrease in the flammability of the epoxy composite [21], which is observed in a decrease in weight loss during ignition in air to 1.8–2.2% and an increase in the oxygen index from 28 to 30–32% by volume, as shown in Table 7. Thus, diorite-filled epoxy composites belong to the class of flame-retardant materials.

Since epoxy resins have good adhesion to most materials [28,29,30], we used the developed compositions for the fire protection of wood. Wood samples with a fire-resistant coating (100 ED-20 + 40 ORFF + 50 Diorite + 15 PEPA) were obtained.

**Figure 7:**
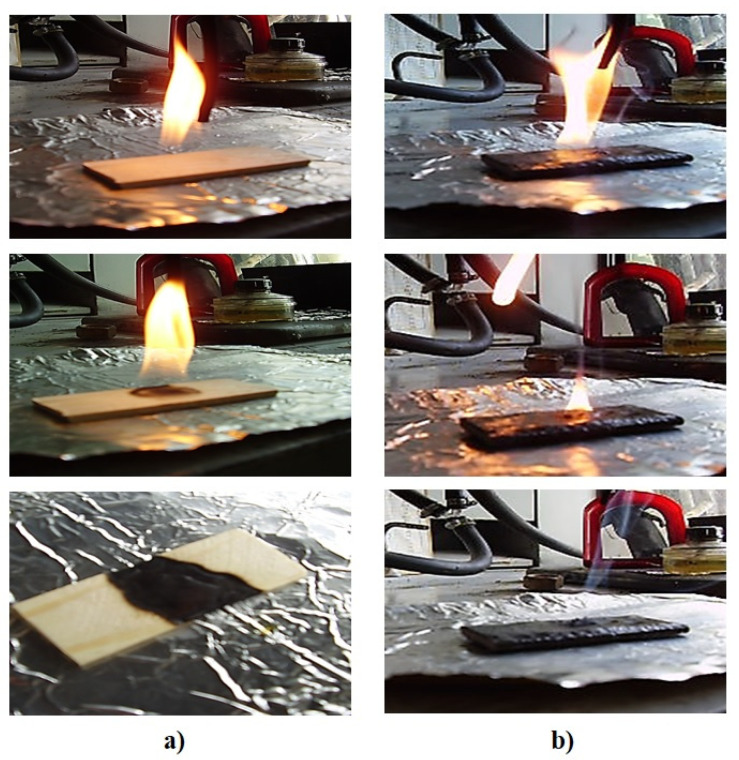
Flame propagation over the wood surface without a fire-resistant coating (**a**) and flame propagation over the wood surface with a fire-resistant coating (**b**).

When determining the speed of flame propagation over the wood surface without a fire-resistant coating and with a fire-resistant coating, it was found that the uncoated wood ignites in 15 s and combustion continues after the gas burner is removed, the flame propagating in the longitudinal and transverse directions at a speed of 30 mm/min, as shown in Figure 7a. Wood with a fire-retardant coating ignites in 45 s; however, the flame extinguishes automatically in 10 s after the gas burner is removed, as shown in Figure 7b.

**Figure 8:**
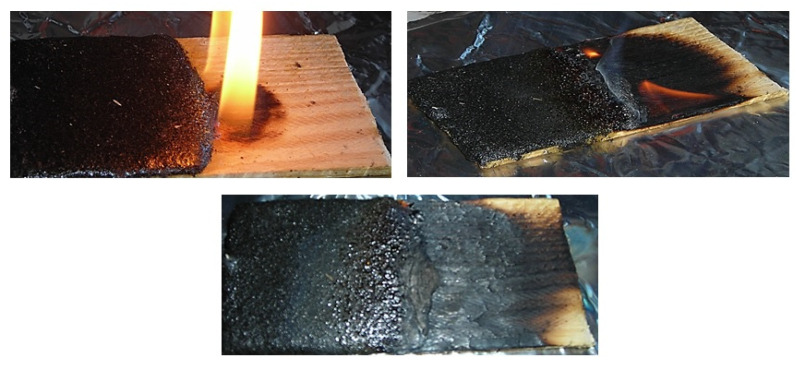
Flame propagation over the surface of a sample with a flame-resistant coating applied only to a part of its surface.

When a sample with a coating applied only to a part of its surface is set on fire, the wood ignites from the uncoated side in 15 s. When the flame comes into contact with the coating, the coating gets foamed, and the flame extinguishes, as shown in Figure 8.

Thus, the possibility of using the developed compositions as fire-resistant coatings for wood has been proven.

**Figure 9:**
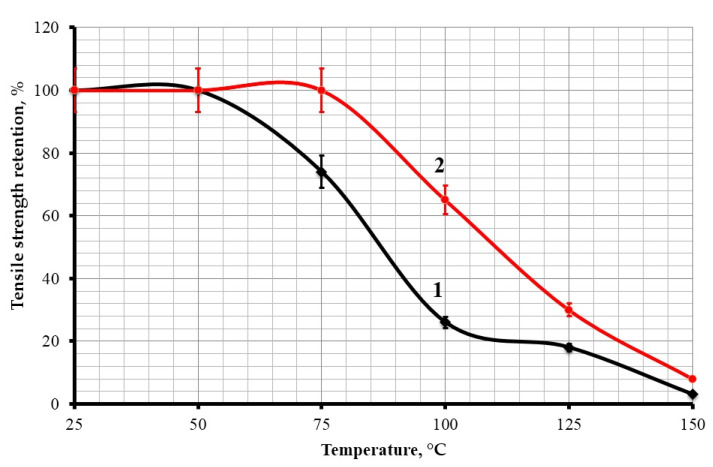
Dependences of the tensile strength index of the epoxy composite on the test temperature: 1—100ED-20 + 40ORPP + 15PEPA; 2—100ED-20 + 40ORPP + 50Diorite + 15PEPA.

The addition of diorite into the epoxy composition leads to an increase not only in thermal resistance, but also to an increase in the thermal stability of the epoxy composite. The studies have shown that the addition of 50 parts by mass of diorite ensures 100% of its tensile strength index at a temperature up to 75 °C, while an unfilled plasticized composite loses about 25% of its tensile strength, as shown in Figure 9.

To reduce the tendency to aggregate and to increase the adhesion capacity of diorite, it is necessary to functionalize it, which should ensure an effective adhesive interaction at the polymer matrix/filler interface. One of the promising methods of functionalization is the surface treatment of fillers with compounds that provide chemical interaction between the filler and the polymer matrix and reduce polydispersity of the filler, which will enhance the physical and mechanical properties of composites based on them [22,31,32,33,34]. APTES was used as such a compound.

**Figure 10:**
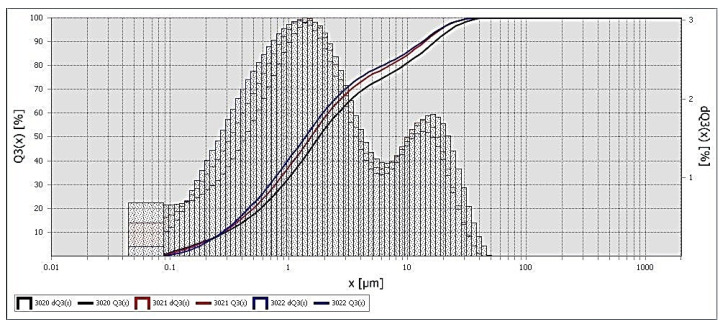
Fractional composition of diorite treated with APTES.

The APTES treatment of the diorite surface changes its fractional composition. An increase in the number of particles with smaller sizes is observed, as shown in Figure 10, as is an increase in the specific surface area of diorite particles from 5.6 to 9.8 m^2^/g.

**Figure 11:**
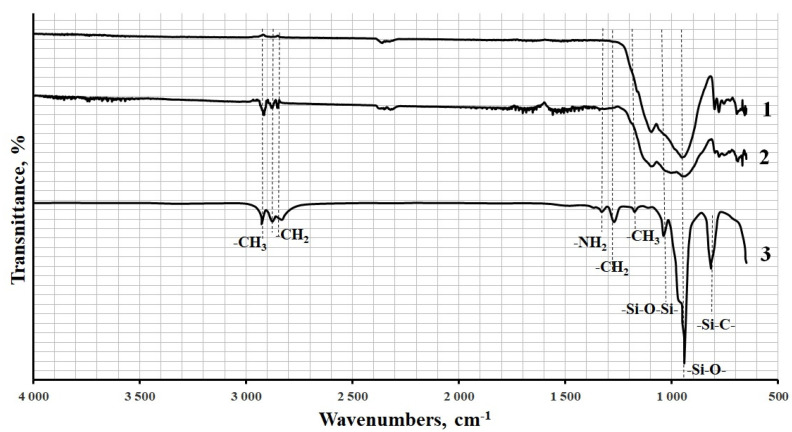
After the APTES treatment of the diorite surface, there appear vibration peaks corresponding to APTES in the IR spectra of functionalized diorite.

The presence of the interaction between the APTES amine groups and the ED-20 epoxy groups was proved earlier [22]. After the APTES treatment of the diorite surface, there appear vibration peaks corresponding to APTES in the IR spectra of functionalized diorite, as shown in Figure 11. Besides, a peak appears at 1040 cm^−1^, which is connected with the formation of a non-hydrolysable -Si-O-Si- bond, which remains after washing the functionalized diorite with water, thus confirming the chemical interaction between the functional groups of APTES and diorite.

The addition of APTES-treated diorite into the epoxy composition accelerates the curing process, which can be seen in the reduction of the duration of the gelation process from 29 to 19 min, and the duration of the curing process from 40 to 28 min, with an increase in the maximum curing temperature from 116 to 122 °C, as shown in Table 5, in comparison with a composition containing original diorite, which further confirms the participation of APTES functional groups in the curing process.

**Figure 12:**
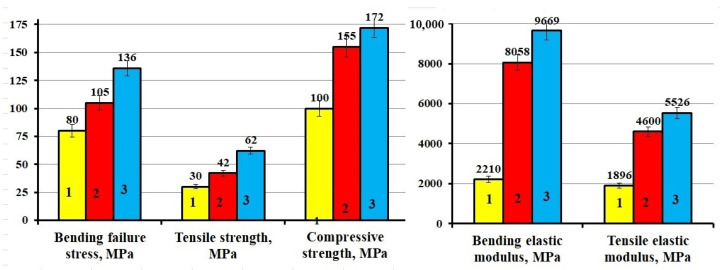
Physicomechanical characteristics of epoxy composites: 1—100 ED-20 + 40 ORPP + 15 PEPA; 2—100 ED-20 + 40 ORPP + 50 Diorite + 15 PEPA; 3—100 ED-20 + 40 ORPP + 50 Diorite_(APTES)_ + 15 PEPA.

The organization of chemical interaction at the polymer matrix/filler interface ensures an increase in the physicomechanical properties of epoxy composites. The addition of APTES-treated diorite into the epoxy composition increases all the studied strength properties by 11–48% as compared to the epoxy composite containing the original diorite, as shown in Figure 12.

Comparison of the developed compositions with existing analogues showed their competitiveness. The developed composites have physicomechanical characteristics comparable to those of the existing analogues and in some cases exceeding them, as shown in Table 8.

## 4. Conclusions

The addition of diorite into the epoxy composition initiates the structure formation processes of an epoxy composite, which is observed in a reduction in the gelation time and the curing time (for a composition with a diorite content of 0.1 parts by mass), wherein the curing time for a composition with a diorite content of 50 parts by mass increases slightly, which is apparently due to the high viscosity of the composition and the steric hindrances of the curing process. Besides, the addition of diorite into the epoxy composition provides an increase in the curing degree of the epoxy composite, which allows the assumption that the diorite particles are additional cross-linking centers.

The addition of diorite into the epoxy composite ensures an increase in the heat resistance, thermal resistance and thermal stability of the composite. The thermal destruction of the epoxy composite containing diorite increases the yield of carbonized structures that prevent the release of volatile pyrolysis products into the gas phase, which reduces the flammability of the epoxy composite. The developed compositions filled with diorite do not support combustion in air and belong to the class of hardly flammable materials.

The efficiency of APTES-functionalization of the diorite surface has been proved, which provides chemical interaction at the polymer matrix/filler interface, and also prevents the aggregation of diorite particles, which, in general, ensures an enhancement of the strength characteristics of epoxy composite materials.

## Figures and Tables

**Table 1:** Typical properties and specifications of epoxy resin, hardener and ORPP.

The Qualitative Characteristics	Value
Properties of epoxy resin ED-20	
Content of epoxy groups, %	20.0–22.5
Viscosity, Pa × s	13–20
Epoxy equivalent, g/mol	195–216
Density at 25 °C, kg/m^3^	1166
Properties of PEPA	
Molecular mass, g/mol	230–250
Viscosity, Pa × s	0.6–0.9
Density at 25 °C, kg/m^3^	1020
Amine number, mg KOH/g	1250
Nitrogen content, % by weight	30.0
Properties of ORPP	
Viscosity, Pa × s	0.4–0.6
Density at 25 °C, kg/m^3^	1250–1295
Phosphorus content, % by weight	10.6–10.8
Acidity, mg KOH/g	0.10–0.12
Boiling temperature, °C	≥300
Decomposition temperature, °C	370

**Table 2:** Chemical composition of diorite.

Component	Concentration, %
Fe_2_O_3_	41.2
SiO_2_	22.9
CaO	20.3
Al_2_O_3_	10.8
TiO_2_	2.4
CuO	1.2
Mn_2_O_7_	0.7
K_2_O	0.3
P	0.1
S	0.06

**Table 3:** Properties of epoxy composites.

Composition, Parts by Mass, Cured by 15 Parts by Mass of PEPA	σ_ben_, MPa	E_ben_, MPa	σ_com_, MPa	σ_ten_, MPa	E_ten_, MPa	a_im_, kJ/m^2^	H_b_, MPa	H_v_, MPa
100ED-20 + 40 ORPP	80 ± 3.2	2210 ± 88	100 ± 4.0	30 ± 1.5	1896 ± 94	6 ± 0.3	175 ± 10	174 ± 10
100 ED-20 + 40 ORPP + 0.05 Diorite	82 ± 3.3	2255 ± 90	105 ± 4.2	40 ± 1.8	2115 ± 105	7 ± 0.4	185 ± 11	184 ± 11
100 ED-20 + 40 ORPP + 0.1 Diorite	95 ± 3.8	2343 ± 92	115 ± 4.5	54 ± 2.4	2490 ± 120	12 ± 0.7	210 ± 12	209 ± 12
100 ED-20 + 40 ORPP + 0.5 Diorite	90 ± 3.5	2506 ± 98	120 ± 4.8	41 ± 1.8	2952 ± 142	7 ± 0.4	240 ± 14	240 ± 14
100 ED-20 + 40 ORPP + 10 Diorite	90 ± 3.6	3556 ± 140	130 ± 5.0	40 ± 1.8	3100 ± 150	5 ± 0.2	250 ± 15	250 ± 15
100 ED-20 + 40 ORPP + 30 Diorite	95 ± 3.7	6540 ± 261	140 ± 5.2	42 ± 2.0	4110 ± 202	5 ± 0.2	275 ± 16	278 ± 16
100 ED-20 + 40 ORPP + 50 Diorite	105 ± 3.9	8058 ± 322	155 ± 6.2	42 ± 2.0	4600 ± 220	5 ± 0.2	295 ± 17	299 ± 17
100 ED-20 + 40 ORPP + 75 Diorite	70 ± 2.6	8950 ± 355	150 ± 6.0	38 ± 1.5	4920 ± 245	4 ± 0.2	310 ± 18	315 ± 18
100 ED-20 + 40 ORPP + 100 Diorite	58 ± 2.1	9812 ± 389	125 ± 4.8	35 ± 1.4	5200 ± 260	4 ± 0.2	340 ± 20	351 ± 20

Note: σ_ben_—bending stress; E_ben_—modulus of elasticity in bending; σ_com_—compressive strength; σ_ten_—tensile strength; E_ten_—tensile modulus of elasticity; a_im_—impact strength, H_b_—Brinell hardness, H_v_—Vickers hardness.

**Table 4:** Viscosity of epoxy compositions at 25 °C.

Composition, Parts by Mass	Viscosity, P × s
100 ED-20 + 40 ORPP	1.50 ± 0.07
100 ED-20 + 40 ORPP + 0.05 Diorite	1.50 ± 0.07
100 ED-20 + 40 ORPP + 0.1 Diorite	1.55 ± 0.08
100 ED-20 + 40 ORPP + 0.5 Diorite	1.60 ± 0.09
100 ED-20 + 40 ORPP + 10 Diorite	3.90 ± 0.18
100 ED-20 + 40 ORPP + 30 Diorite	15.20 ± 0.65
100 ED-20 + 40 ORPP + 50 Diorite	24.50 ± 1.10
100 ED-20 + 40 ORPP + 75 Diorite	32.30 ± 1.40
100 ED-20 + 40 ORPP + 100 Diorite	40.60 ± 1.80

**Table 5:** Values of the curing process of epoxy composites.

Composition, Parts by Mass, Cured by 15 Parts by Mass of PEPA	τ_gel_, min	τ_cur_, min	T_max_, °C	X, %
100 ED-20 + 40 ORPP	27	38	88	90.0
100 ED-20 + 40 ORPP + 0.1 Diorite	24	34	135	94.0
100 ED-20 + 40 ORPP + 50 Diorite	29	40	116	98.0
100 ED-20 + 40 ORPP + 50 Diorite_(APTES)_	19	28	122	98.6

Note: τ_gel_—the duration of gelation process, τ_cur_—the duration of curing, T_max_—the maximum curing temperature, X—curing degree.

**Table 6:** Results of differential scanning calorimetry of epoxy compositions.

Composition, Parts by Mass, Cured by 15 Parts by Mass of PEPA	T_start_–T_end_ T_max_ °C	T_glass_, °C	H, J/g
100 ED-20 + 40 ORPP	46–167 108	78	415
100 ED-20 + 40 ORPP + 0.1 Diorite	36–175 110	81	893
100 ED-20 + 40 ORPP + 50 Diorite	34–211 109	84	703

Note: T_start_, T_end_—temperature of the start and end of the curing process, T_max_—the temperature of the maximum heat release during curing, H—thermal effect of reaction.

**Table 7:** Physicochemical properties of epoxy composites.

Composition, Parts by Mass, Cured by 15 Parts by Mass of PEPA	T_in_, °C	T_f_ °C	Yield of CS at T_f_, % Mass	Δm, %	OI, %	T_v_, °C
100 ED-20	200	390	40 (390 °C)	78	19	86
100 ED-20 + 40 ORPP	230	370	54 (370 °C)	4.7	28	132
100 ED-20 + 40 ORPP + 0.1 Diorite	240	380	56 (380 °C)	4.4	28	140
100 ED-20 + 40 ORPP + 50 Diorite	245	380	70 (380 °C)	2.2	30	180
100 ED-20 + 40 ORPP + 100 Diorite	245	370	77 (370 °C)	1.8	32	188

Note: T_in_, T_f_—initial and final temperature of the main stage of thermolysis; Δm—weight loss when ignited in air; OI—oxygen index; T_v_—Vicat heat resistance.

**Table 8:** Comparison of the developed materials with analogues.

Composition	σ_ben_, MPa	E_ben_, MPa	σ_com_, MPa	σ_ten_, MPa	E_ten_, MPa
ED-20 + ORPP + PEPA + Diorite_(APTES)_	136	9669	172	62	5526
Analogs					
Epoxy resin + TETA + Fly ash [16]	-	-	160	48	-
DER-331 + JOINTMINE 905-3s + MMT [18]	-	-	-	50	1500
ED-20 + ORPP + PEPA + Ocher [7]	102	10,163	156	45	4110
ED-20 + PEPA + UDD [4]	102	3200	-	-	-
Epoxy resin + TETA + Granite_(TEMS)_ [19]	79	5126	107	-	-

Note: σ_ben_—bending stress; E_ben_—modulus of elasticity in bending; σ_com_—compressive strength; σ_ten_—tensile strength; E_ten_—tensile modulus of elasticity; TETA—triethylenetetramine; DER-331—diglycidyl ether of bisphenol-A epoxy resin; JOINTMINE 905-3s—modified cycloaliphatic amine hardener; MMT—bi-functionalized montmorillonite; UDD—ultra-dispersed diamond; TEMS—triethoxymethylsilane.

## Data Availability

The data presented in this study are available on request from the corresponding author.
